# How deadly is a fracture distal to the hip in the elderly? An observational cohort study of 11,799 femoral fractures in the Swedish Fracture Register

**DOI:** 10.1080/17453674.2020.1831236

**Published:** 2020-10-26

**Authors:** Olof Wolf, Sebastian Mukka, Jan Ekelund, Michael Möller, Nils P Hailer

**Affiliations:** a Department of Surgical Sciences, Orthopaedics, Uppsala University, Uppsala;; b Department of Surgical and Perioperative Sciences at Umeå University, Umeå;; c Centre of Registers Västra Götaland, Gothenburg;; d Institute of Clinical Sciences, Sahlgrenska Academy, University of Gothenburg, Gothenburg, Sweden

## Abstract

Background and purpose — Unlike hip fractures, diaphyseal and distal femoral fractures in elderly patients have not been widely studied. We investigated the demographics, comorbidities and mortality of patients with femoral fractures at any anatomical level with a focus on early mortality.

Patients and methods — We analyzed 11,799 patients ≥ 65 years with a femoral fracture registered in the Swedish Fracture Register from 2011 to 2014. The cohort was matched with the National Patient Register to obtain data on comorbidities classified according to the Charlson Comorbidity Index (CCI). Generalized linear models were fitted to estimate the adjusted relative risk of mortality.

Results — Mean age of the cohort was 83 years and 69% were women. Patients with distal femoral fractures had the lowest degree of comorbidity, with 9% having a CCI of ≥ 3 compared with 14% among those with proximal and 16% among those with diaphyseal fractures. Unadjusted 90-day mortalities were 13% (95% CI 9.4–16) after fractures in the distal, 13% (CI 10–16) in the diaphyseal, and 15% (CI 14–15) in the proximal segment. The adjusted relative risk for 90-day mortality was 1.1 (CI 0.86–1.4) for patients with distal and 0.97 (CI 0.76–1.2) for patients with diaphyseal femoral fractures when compared with patients with hip fractures.

Interpretation — Elderly patients with femoral fractures distal to the hip may have similar adjusted early mortality risks to those with hip fractures. There is a need for larger, preferably prospective, studies investigating the effect of rapid pathways and geriatric co-management for patients with diaphyseal and distal femoral fractures.

1-year mortality after proximal femur fractures is up to 30% and is higher in men (Do et al. 2016, Mattisson et al. 2018). Proximal femoral fractures have been thoroughly investigated for outcomes after different treatment modalities (Gdalevich et al. 2004, Sircar et al. 2007, Al-Ani et al. 2008, Khan et al. 2009, Hansson et al. 2017, Bartels et al. 2018, Dolatowski et al. 2019).

In contrast, little research has been conducted on femoral fractures distal to the proximal segment in the elderly population. However, there is some evidence to suggest that patients with diaphyseal and distal femur fractures have similar mortality and mobility risks to those with proximal femur fractures (Konda et al. 2015, Myers et al. 2018, Larsen et al. 2020). Elderly patients with fractures of the femur distal to the hip also seem to be similar to hip fracture patients in age and sex distribution. However, there is little information on their degree of comorbidities (Smith et al. 2015) and, to our knowledge, no comparative study has been performed on mortality after femoral fractures distal to the hip. Consequently, guidelines are lacking on the treatment of elderly patients with diaphyseal and distal femoral fractures.

The Swedish Fracture Register (SFR) was launched in 2011 to prospectively monitor fracture treatment performed in Sweden and collect information on all fractures, including data on injury mechanisms, fracture characteristics, and treatments (Wennergren et al. 2015). Data from this register has been matched with the National Patient Register (NPR) to obtain information on comorbidities and causes of death (Ludvigsson et al. 2009). We thus designed an observational study on a cohort of elderly patients with femoral fractures with the primary aim to evaluate the association of fracture localization with mortality, adjusting for pre-existing comorbidities.

## Patients and methods

### Study design and variables

We designed an observational cohort study of patients with femoral fractures. The treating orthopedic surgeon registers all Swedish patients treated for any fracture at departments affiliated to the SFR. The SFR started with registration in Gothenburg only in 2011. In 2014, the SFR had a coverage of 40% of the orthopedic departments, which gave us data from 24 hospitals, and by 2020 all orthopedic departments in Sweden will be active in the SFR. Details are collected on injury mechanisms, trauma energy content, fracture type according to the AO/OTA classification, time and date of radiography, and details on treatment, including the type of osteosynthesis or joint replacement and the surgeon’s level of expertise (Wennergren et al. 2015). Information on mortality is obtained by real-time linkage to the Swedish National Death Register. This cohort was matched with the NPR to collect data on comorbidities and causes of death. The Charlson Comorbidity Index (CCI), modified according to Quan et al. (2011), was calculated from ICD codes 12 months before the date of injury and categorized into 3 groups: CCI = 0, CCI = 1–2, and CCI = ≥ 3, representing no, moderate, and high comorbidity, respectively. We followed the STROBE guidelines for the reporting of this observational study.

### Patient selection

**Figure 1.**
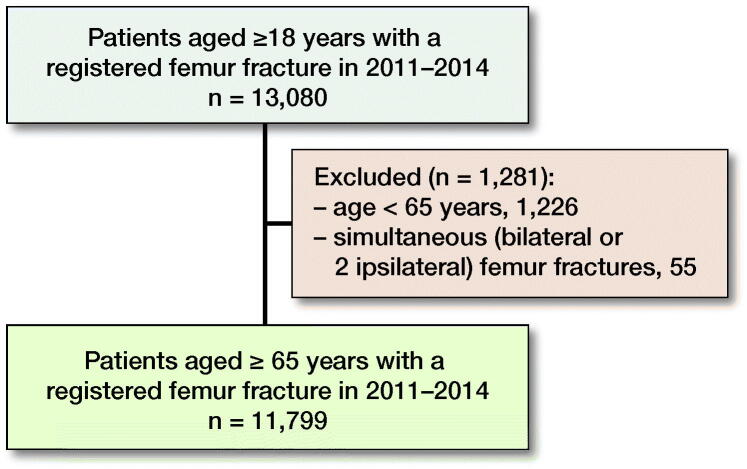
Flowchart of patients included in the study.

We retrieved information on all patients aged ≥ 65 years registered with a femoral fracture (International Classification of Diseases [ICD] S72.0–S72.4) between January 1, 2011 and December 31, 2014. To avoid dependency issues, we included only data concerning the first fracture in patients with a subsequent femoral fracture during the study period. For the same reason, patients with simultaneous bilateral or simultaneous fractures at several anatomical levels were excluded (Figure 1). Patients with periprosthetic (after hip or knee arthroplasty) and implant-related (after previous plate, nail, or screw) fractures were included in the main analysis but were also analyzed separately.

### Outcome measures

We analyzed (1) the adjusted relative risk (RR) of 90-day mortality dependent on fracture location (proximal, diaphyseal, or distal part of the femur), (2) the adjusted RR of 30- and 365-day mortality dependent on fracture location as above, (3) the association between age, sex, and pre-existing comorbidities and mortality, and (4) the distribution of fracture location and mortality of patients with subsequent fractures, periprosthetic, or implant-related fractures.

### Statistics

Baseline variables are presented as means (SDs) and proportions, cross-tabulated by femoral segment. Differences between observed counts were analyzed using the chi-square test. Unadjusted cumulative mortality was estimated using the Kaplan–Meier method. Generalized linear models with binomial distribution and a log-link were fitted to estimate the RR of 30-, 90-, and 365-day mortality by fracture location, adjusted for age, sex, and CCI with 95% confidence intervals (CI). Follow-up mortality data was retrieved for 1 year for all patients. Statistical analyses were performed using IBM SPSS Statistics, version 26 for Mac (IBM Corp, Armonk, NY, USA) and SAS, version 9.4 (SAS Institute, Cary, IN, USA). Survival curves with CIs were plotted using the R software package (R Development Core Team 2017).

### Ethics, funding, and potential conflicts of interest

The study was conducted following the ethical principles of the Helsinki Declaration and was approved by the Regional Ethical Committee in Uppsala (Dnr 2015/510; date of approval January 20, 2016). In accordance with Swedish law, individual consent was not required. This study was supported by Stiftelsen Skobranschens Utvecklingsfond and by the Swedish Research Coucil (VR 2018–02612). The authors declare no competing interests.

## Results

### Characteristics of the study population

The final study cohort comprised 11,799 patients with a femoral fracture (Figure 1, Table 1). The mean age of the patients was 83 years (SD 8) and 69% were women. Of the fractures, 3% occurred in the distal, 4% in the diaphyseal, and 93% in the proximal femur. A same-level fall was the cause of injury in 93% of all patients, 3.6% fell from another level, and 2% of the injuries were traffic-related. Most fractures (97%) were from a low-energy injury, and 0.3% (n = 31) were open fractures.

**Table 1.** Baseline demographic characteristics of the study population of 11,799 patients with femoral fractures. Values are distribution of the Charlson Comorbidity Index category and subcategories (%) depending on fracture segment

Description	Proximal	Diaphyseal	Distal
Distribution, n (%)	10,964 (93)	469 (4)	366 (3)
Mean age (SD)	83 (8)	82 (9)	82 (9)
Sex, n (%)			
Male	3,441 (31)	141 (30)	68 (19)
Female	7,523 (69)	328 (70)	298 (81)
CCI ^a^			
0	54	54	63
1–2	33	29	28
3 or more	14	16	9
Charlson subcategories ^a^			
Myocardial infarction	9.4	8.3	6.6
Congestive heart failure	13	11	13
Peripheral vascular disease	3.3	1.5	1.9
Cerebrovascular disease	11	8.7	9.8
Dementia	17	11	7.4
Chronic pulmonary disease	10	11	6.6
Rheumatic disease	3.8	6.4	5.7
Peptic ulcer disease	1.0	0.6	0.5
Mild liver disease	0.7	0.0	1.4
Diabetes without chronic			
complications	13	10	9.3
Hemiplegia or paraplegia	1.6	1.3	1.6
Renal disease	4.6	3.4	5.7
Diabetes with chronic			
complications	2.3	2.3	3.0
Malignancy	8.9	12	4.6
Moderate or severe liver			
disease	0.2	0.2	0.3
Metastatic solid tumor	2.3	7.5	1.1
Aids/HIV	0	0	0

**^a^** Proportion (%) within segment

The proportion of patients with high comorbidity (CCI ≥ 3) was lower among patients with distal fractures (9%) when compared with patients with diaphyseal (16%) or proximal fractures (14%, Table 1). When stratified by sex, men had similar comorbidity patterns independent of the femoral fracture segment. Women with distal femoral fractures had a similar proportion of high comorbidity (8.7%) compared with those with proximal fractures (11%), whereas women with diaphyseal fractures had a higher proportion of high comorbidity (17%) compared with proximal fractures. For men, 19% had high comorbidity compared with 11% of the women. Finally, 48% of the men and 57% of the women had no comorbidity (Table 1).

### 90-day mortality

**Figure 2.**
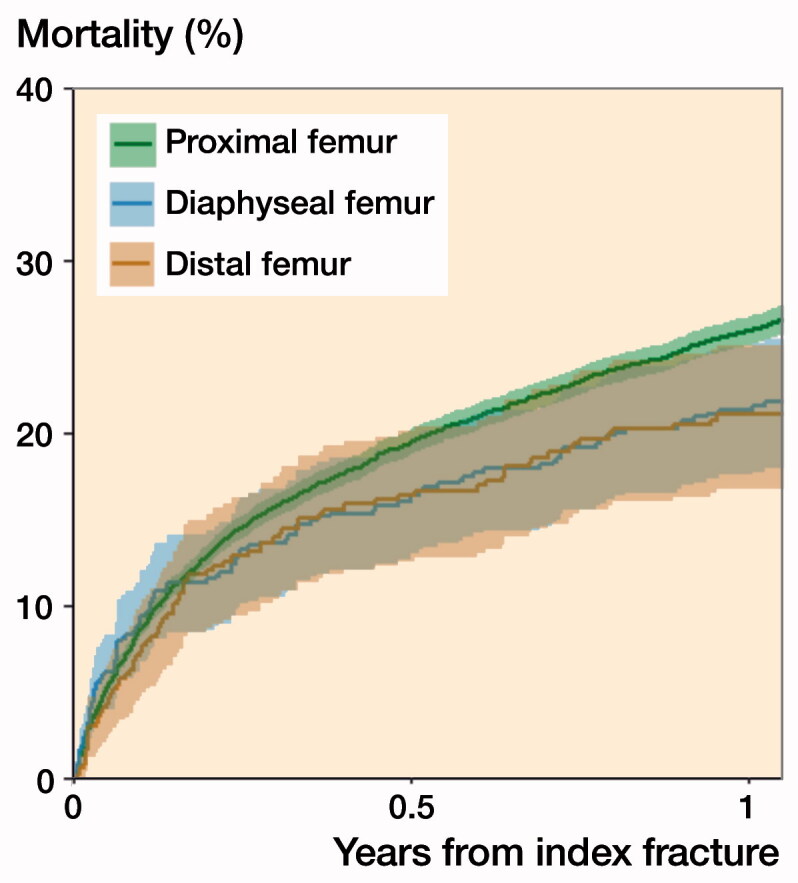
Unadjusted cumulative mortality up to 1 year after index fracture per femoral segment with 95% confidence intervals.

The unadjusted 90-day mortality rates were 13% (CI 9.4–16) for patients with distal, 13% (CI 10–16) for those with diaphyseal, and 15% (CI 14–15) for those with proximal segment fractures (Figure 2). We found no statistically significant difference in the adjusted RR of 90-day mortality when dividing patients by fracture location although confidence intervals were wide due to the size of the sample: RR = 1.1 (CI 0.9–1.4) for patients with distal and RR = 1.0 (CI 0.8–1.2) for patients with diaphyseal fractures when compared with patients with proximal femoral fractures. No statistically significant differences in the adjusted mortality risk among patients with the 3 different fracture locations were observed when stratifying the analyses by sex or CCI group (Table 2).

**Table 2.** Unadjusted 90-day mortality dependent on fracture location and adjusted for age, sex, and the Charlson Comorbidity Index (CCI), including stratified analyses for sex and CCI groups. Values are percentage and relative risks (RR) with 95% confidence intervals (CI)

Fracture location	Mortality (%)	RR (CI)
Unadjusted 90-day mortality		
Proximal	15 (CI 14–15)	
Diaphyseal	13 (CI 10–16)	
Distal	13 (CI 9.4–16)	
Proximal		1 ref
Diaphyseal		1.0 (0.8–1.2)
Distal		1.1 (0.9–1.4)
90-day mortality when stratified by sex		
Men		
Proximal		1 ref
Diaphyseal		1.2 (0.8–1.6)
Distal		1.1 (0.6–1.7)
Women		
Proximal		1 ref
Diaphyseal		0.8 (0.6–1.1)
Distal		1.2 (0.9–1.5)
90-day mortality when stratified by CCI		
No (CCI = 0)		
Proximal		1 ref
Diaphyseal		0.8 (0.5–1.2)
Distal		0.9 (0.5–1.3)
Moderate (CCI = 1–2)		
Proximal		1 ref
Diaphyseal		0.8 (0.5–1.2)
Distal		1.4 (1.0–2.0)
High (CCI = 3 or more)		
Proximal		1 ref
Diaphyseal		1.2 (0.8–1.6)
Distal		1.1 (0.6–1.7)

### 30-day and 1-year mortality

After 30 days, the unadjusted cumulative mortality was 6.3% for patients with distal, 8.3% for those with diaphyseal, and 7.5% for those with proximal femoral fractures. The unadjusted cumulative mortalities after 1 year were 21% for patients with distal, 21% for those with diaphyseal, and 26% for those proximal femoral fractures (Table 3). We found no statistically significant differences in the adjusted risk of 30-day or 1-year mortality among patients with these fracture locations.

**Table 3.** Unadjusted 30- and 365-day mortality dependent on fracture location. Values are mean percentage (95% CI)

Fracture location	30-day	365-day
Proximal	7.5 (7.0–8.0)	26 (25–27)
Diaphyseal	8.3 (5.8–11)	21 (18–25)
Distal	6.3 (3.8–8.8)	21 (17–25)

### Subsequent fractures

During the study period, 3% (n = 350) of the patients had a subsequent femoral fracture of any kind. Of these, no patients with an initial distal or diaphyseal and 2 patients with an initial proximal femoral fracture died within 30 days of the first fracture. Comparing patients with a subsequent femoral fracture of any kind by initial fracture locations, the unadjusted 90-day mortality rates were thus 0% for patients with distal, 7.7% (CI 0–22; n = 1 of 13) for those with diaphyseal, and 5.8% (CI 3–8; n = 19 of 329) for those with proximal fractures compared with13%, 13%, and 15%, respectively, for patients without subsequent fractures. Likewise, the 1-year cumulative mortality of patients sustaining a subsequent femoral fracture was 13% for those with a distal, 15% for those with a diaphyseal, and 19% for those with a proximal femoral fracture.

### Periprosthetic and implant-related fractures

3.2% (n = 383) of all fractures were periprosthetic. Of all periprosthetic fractures, 29% were in the distal, 47% in the diaphyseal, and 24% in the proximal segment. 30% of all distal fractures, 38% of all diaphyseal, and 1% of all proximal fractures were periprosthetic. The 1-year cumulative mortality was 19% for patients with distal, 18% for patients with diaphyseal, and 19% for those with proximal femoral periprosthetic fractures.

1.2% (n = 137) of the cohort had implant-related femoral fractures. Of all implant-related fractures, 20% were in the distal, 45% in the diaphyseal, and 36% in the proximal femur. 7% of all patients with distal fractures, 13% of those with diaphyseal, and 0.4% of those with proximal fractures had implant-related fractures. The 1-year cumulative mortality was 19% for patients with distal, 12% for those with diaphyseal, and 18% for those with implant-related femoral fractures.

## Discussion

In this observational cohort study we find that elderly patients with a femoral fracture at the distal or diaphyseal level have a similar adjusted 90-day mortality risk to hip fracture patients. Patients with a distal femoral fracture are less comorbid than those with a proximal fracture, and there is a higher proportion of women among these than in patients with diaphyseal or proximal fractures.

We are not aware of any other comparative study on femoral fractures examining the effect of fracture location on mortality. Some studies have investigated the mortality of patients with distal femoral fractures and compared those with previously reported figures on hip fracture mortality (Nyholm et al. 2017, Larsen et al. 2020). The 1-year mortality was 35% for patients > 60 years compared with 3% in those who were < 60 years in a study on distal femoral fractures (Larsen et al. 2020). Our figures on mortality after distal femoral fractures correspond well with the mortality rates of 7% at 30 days and 18% at 1 year found in a retrospective study from the UK in 105 patients > 50 years (Smith et al. 2015). Another study from the Danish Fracture Database on 392 patients > 50 years with a closed low-energy distal femoral fracture reported mortality rates of 7.1% at 30 days and 13% at 90 days (Nyholm et al. 2017). Patients with a periprosthetic distal femoral fracture and patients with comorbidities exhibit higher mortality rates (Streubel et al. 2011, Kammerlander et al. 2012). In addition, an increasing ASA score and male sex are associated with higher mortality in both proximal and distal femoral fractures (Nyholm et al. 2015, 2017).

Half of the periprosthetic fractures occurred in the femoral diaphysis and the remaining half were evenly distributed between the proximal and distal femoral segments. Approximately a third of the distal and diaphyseal fractures were periprosthetic and these are often amenable to fracture fixation as opposed to the proximal periprosthetic fractures. A loose total hip arthroplasty requires extensive revision surgery and such surgery has been associated with high mortality and an overall complication rate of 18% (Lindahl et al 2005, Marsland and Mears 2012). In contrast, we found no excessive mortality rates in the groups of patients with periprosthetic or implant-related femoral fractures.

A subsequent femoral fracture would at first thought be expected to enhance mortality. Our opposite finding of lower mortality rates in patients with subsequent fractures may be due to immortal time bias introduced by the fact that patients with second fractures must have survived their first fracture.

Dementia is common in hip fracture patients (Friedman et al. 2010) and has been associated with higher mortality, reduced walking ability, and lower recovery to full ADL function after a hip fracture (Larsson et al. 2019, Delgado et al. 2020). We found a higher proportion of dementia cases among patients with a proximal femoral fracture compared with patients with fractures further distal in the femur. Although there was a difference in the distribution of dementia between fracture locations, there was no association with mortality. Patients with pre-fracture dementia have a higher risk of developing delirium, which must be accounted for in the care of hip fracture patients (Krogseth et al. 2016). Our rates of dementia were substantially lower than the 50% of hip fracture patients included in an RCT evaluating orthogeriatric care on cognitive function following hip fracture (Watne et al. 2014), but this may be due to more stringent analysis of this specific comorbidity in the cited RCT.

With over 90% of the femoral fractures in the elderly being proximal femoral fractures, mortality rates have been thoroughly investigated. Actions such as multi-professional or orthogeriatric care and hip fracture pathways have been effective in reducing mortality rates and improving functional outcomes (Pedersen et al. 2008, Adunsky et al. 2011, Leung et al. 2011, Prestmo et al. 2015, Mukherjee et al. 2020). Pathways without geriatric involvement have failed to show beneficiary results (Haugan et al. 2017, Svenoy et al. 2020). Moreover, surgery on hip fractures within 24 hours has been advocated to decrease complications and lower mortality rates (Nyholm et al. 2015). By contrast, a delay of up to 48 hours post-admission does not affect mortality according to a cohort study of over 70,000 patients from the Norwegian Hip Fracture Register (Leer-Salvesen et al. 2019). Elderly patients with hip fractures have higher mortality risk following surgical delay, a risk that is enhanced in men and patients with multiple comorbidities (Beaupre et al. 2019). Greve et al. (2020) reported higher mortality in patients with an ASA score of 3–4, but surprisingly also in women, when surgical waiting time was > 24 hours from admission. All this has led to an effort in many countries to operate hip fractures within 24 hours—or as the NICE guidelines in the UK say, “perform surgery on the day of, or the day after, admission” (National Institute for Health and Care Excellence 2011). Conflicting results from 2 studies on surgical delay and the effect on mortality after proximal (Nyholm et al. 2015) and distal femoral fractures (Nyholm et al. 2017) have been reported from the Danish Fracture Database. In these stydies delay had no effect on mortality after distal femoral fractures but a significant effect already after a 12-hour delay for proximal femoral fractures. This difference may be attributed to the fact that 80% with distal femoral fractures were women compared with 70% with proximal fractures (and diaphyseal in our findings; Konda et al. 2015, Nyholm et al. 2015, 2017). Ultra-fast surgery after hip fracture indicated no measurable effect on mortality or major complications in the HIP ATTACK trial (HIP ATTACK Investigators 2020).

Of note, a higher level of surgeon expertise was associated with decreased mortality for proximal but not for distal femoral fractures (Nyholm et al. 2015, 2017). Displaced femoral neck fractures are routinely operated on with arthroplasty, which is uncommon for complex and comminuted fractures in the distal part of the femur. However, most distal femoral fractures are extraarticular or have a simple articular component and should be amenable to routine periarticular plating or nailing without delay (Nyholm et al. 2017). The few patients needing complex reconstruction by a specialized orthopedic trauma surgeon should not dictate the routine for the majority.

This study has several limitations, most of which are due to the register-based observational design. Time to surgery was added to the SFR in late 2014 and is a crucial variable in evaluating mortality in elderly patients with femoral fractures. Further research with newer data could provide information on the impact of preoperative waiting time on mortality by fractured femoral segment. The majority (93%) of patients in our cohort had proximal femoral fractures. However, with a study population of almost 12,000 patients, the distal femoral fracture group is larger than in some previous studies (Kammerlander et al. 2012, Smith et al. 2015, Nyholm et al. 2017, Myers et al. 2018, Larsen et al. 2020). We also analyzed elderly patients with diaphyseal fractures, which allowed us to compare all femoral fractures in the elderly patient. We lack information on function level and the ASA score. Such information would strengthen our comparisons and conclusions; however, the CCI retrieved from the NPR gives us a good indication of the comorbidity of the patients. Given the nature of register-based research, we have retrieved a large cohort of femoral fractures registered and classified by the treating surgeons. Such an approach increases the generalizability of our results compared with a single-center retrospective study although the data in this study was retrieved in an early phase of the SFR with 40% of the orthopedic departments taking active part in the registration. We assessed a cohort of > 11,000 femoral fractures and retrieved data for comorbidity for an adjusted analysis. Data on fracture localization affecting mortality was adjusted for age, sex, and comorbidity as assessed by the CCI. The NPR has been validated previously (Ludvigsson et al. 2011) and there is recurrent work to audit the completeness of data in the SFR. For hip fractures, most of the hospitals have completeness of registration > 90% compared with the NPR.

To conclude, after adjustment for sex, age, and comorbidities femoral fractures in the elderly seem to be equally deadly independent of the anatomical segment that is injured. Our interpretation may be hampered by the limited precision of our risk estimates that leaves room for type II errors. There is a need for larger, preferably prospective, studies investigating the effect of rapid pathways, early surgery, and geriatric co-management also for patients with diaphyseal and distal femoral fractures.
